# Does preoperative analysis of intrahepatic venous anastomoses improve the surgeon's intraoperative decision making? Pilot data from a case report

**DOI:** 10.1186/1754-9493-2-19

**Published:** 2008-08-21

**Authors:** Lars Fischer, Max Schoebinger, Jan-Oliver Neumann, Sascha Müller, Hans-Peter Meinzer, Markus W Büchler, Bruno M Schmied

**Affiliations:** 1 Department of Surgery, University of Heidelberg, INF 110, D-69120 Heidelberg, Germany; 2 DKFZ (German Cancer Research Center) Heidelberg, INF 280, D-69120 Heidelberg, Germany

## Abstract

**Background:**

Three-dimensional (3D) visualization is thought to improve the anatomical understanding of clinicians, thus improving patient safety.

**Case presentation:**

A 58-year-old male was admitted to our hospital in April 2007 with a suspected metastasis of a sigmoid cancer in the Couinaud segment (CS) 7. The anatomical situation of this patient was analyzed using both a CT scan and 3D images. The initial CT scan revealed that the proximal part of the middle hepatic vein was completely missing and the metastasis in the CS 7 was closely attached to the right hepatic vein. After analyzing additional 3D images, it became clear that due to the close proximity of metastasis and right hepatic vein, the resection of the right hepatic vein was inevitable. Based on this 3D analysis, it was decided to perform a right-sided hemihepatectomy. In this case report, 3D visualization resulted in a faster and clearer understanding of the unique anatomical situation in a patient with complicated liver anatomy than transversal CT images.

**Conclusion:**

The here presented data shows for the first time 3D visualization of intravenous anastomoses in the human liver. The information offered by 3D visualization is not redundant but rather serves as a true source of additional information, indicating the potential benefit of 3D visualization in surgical operation planning.

## Background

Three-dimensional (3D) visualization is thought to ameliorate anatomical understanding among surgeons as well as medical students [1-3] Better perception of anatomy improves the surgeon's ability to accurately plan and perform surgical procedures [1,4], an effect which can result in lower morbidity and mortality rates after surgical interventions.

Our group has been working on 3D operation planning in liver surgery for many years [5-8]. Three-dimensional visualization based on CT scans is an efficient and fast tool for analyzing liver anatomy, possible resection proposals and volumetric consequences of the planned resections [5,9,10]. Particularly, the assessment of post-resectional functioning liver parenchyma is an important issue in planning of liver resections. Is has been shown that computer-assisted operation planning has a potential use of for assessment of functional respectability [11].

The existence of intrahepatic venous anastomoses (IVA) between the main stems of the hepatic veins (i.e. middle, right, and left) has been known for many years [12,13]. Couinaud [12] found anastomoses between intrahepatic veins in 25 out of 30 casts and such anastomoses must be considered a reality from the anatomical point of view [13]. The question whether these IVA exist only in pathological livers or also reflect physiological vascular patterns has been discussed heatedly from the beginning [13,14]. However, there are a few case reports describing the importance of such anastomoses under clinical conditions [15-18].

To our knowledge, this is the first published report of 3D visualization of intravenous anastomoses in the human liver. Because 3D imaging leads to a faster and easier understanding of the individual anatomical situation, it may be an appropriate tool to potentially increase patient safety not only in visceral surgery but also in other surgical areas [19-22].

## Case presentation

### Description of the patient

**Figure 1:**
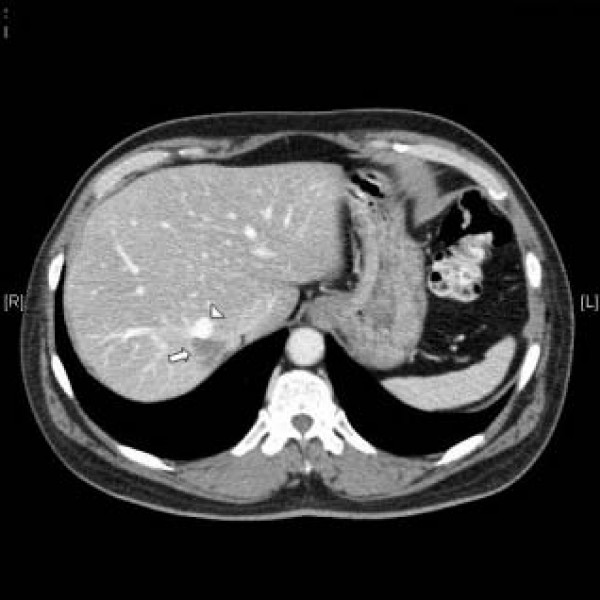
Transversal CT image of the patient's liver showing the right (arrowhead) in proximity to the tumor (arrow).

**Figure 2:**
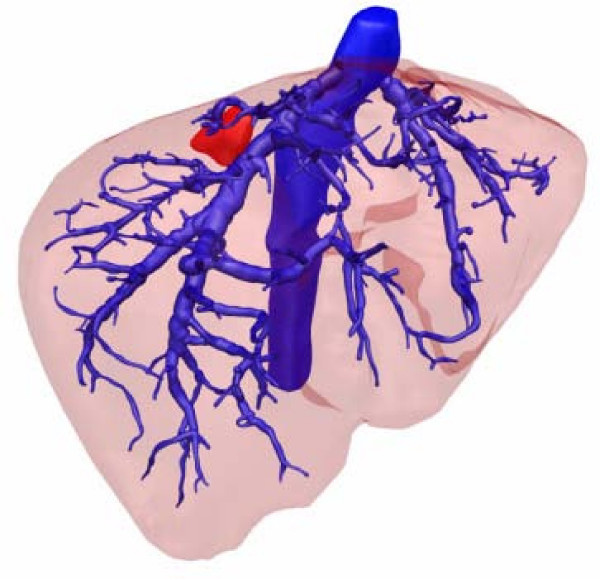
**3D visualization of the venous anatomy. **The observer has a view from above the liver. Here, the proximal part of the middle hepatic vein is missing. The tumor is colored in red.

A 58-year-old male was admitted to our hospital in April 2007 with a suspected metastasis of a sigmoid cancer in the Couinaud segment (CS) 7 found during a follow-up examination using ultrasound. The patient had undergone a resection of the colon sigmoideum due to carcinoma in 2003. In addition to the liver metastasis, the patient suffered from mild hypertension and non-insulin-dependent diabetes mellitus. The only preoperative imaging was an abdominal ultrasound; thus, an additional CT scan of the abdomen was performed in preparation for the surgical intervention. The metastasis in the CS 7 had a size of 2.2 × 2.4 cm and was closely attached to the right hepatic vein (Figure 1). After careful analysis of the CT scan, it became clear that the proximal part of the middle hepatic vein was completely missing, whereas its distal branches seemed to be open. In order to get more detailed information about the anatomical situation, an additional 3D imaging of this case was performed. It was evident that the middle hepatic vein was missing. Most likely this anatomical pattern was not tumour related because there was a close proximity of metastasis and right hepatic vein (Figure 2) making the resection of the right hepatic vein inevitable. As the non-existence of a middle hepatic vein may have led to insufficient drainage of remnant hepatic parenchyma following a bisegmental resection of CS 6 and 7, it was decided to perform a right-sided hemihepatectomy. The pathologic examination confirmed the diagnosis of a colorectal liver metastasis. The surrounding liver parenchyma did not show any distinctive features, and in particular, no evidence of liver cirrhosis. The intra- and postoperative courses of the patient were uneventful. The patient was discharged on the eighth postoperative day.

### CT scans

The according CT scan was made available by the Radiology Department of the German Cancer Research Center, Heidelberg. The image data was acquired preoperatively during the routinely performed CT scan.

### Image data

Image data were acquired with a Toshiba Aquilion 16 slice multidetector CT scanner (Toshiba, Japan). A standard bi- or triphasic liver scan with an optimized portal venous phase was performed. SureStart bolus tracking technique (130 ml Imeron 300, Altana, Germany, flow rate 4–5 ml/s) was used in all protocols to optimize vascular contrast [26].

### Data transfer and digital postprocessing

Imaging data sets were transferred to the Department of Medical and Biological Informatics of the German Cancer Research Center using the CHILI [23] teleradiology system (CHILI GmbH, Heidelberg, Germany). Segmentation of the liver was performed using interactive region growing techniques as previously described by our group [6]. The vessels were segmented using a grey-value-based volume-growing technique. The segmentation was then transformed automatically into a symbolic representation of the vascular anatomy containing the vessel paths, and locations of bifurcations as well as the vessels diameters [24]. The origin of the left, middle, and right hepatic veins were identified, clicked upon and all depending vessels were marked automatically as branches of that particular vein. Portal veins and arteries were defined in the same manner. All results were validated by a team consisting of surgeons, radiologists and medical computing specialists.

Since the whole process from CT data to 3D viszualization preserves all positional information, 3D visualization and original CT data can be presented in a consistent manner. Several tools are available for the interactive exploration of the individual patient anatomy. One of these tools, the "re-localization tool", allowed us to define any volume element (voxel) in the CT scan and find this particular voxel within the 3D visualization. This is of special importance because the localization of intrahepatic venous shunts is more easily achieved with the help of 3D images.

## Conclusion

Even though 3D visualization is now available for a couple of organ systems, the technique must still be described as rather new [25-27]. It has been shown in the field of liver surgery that 3D visualization leads to a better understanding of the liver anatomy and in turn improves surgical operation planning [1,2]. In order to optimize the conception of the anatomical situation and thereby potentially improve patient safety, we routinely perform 3D analyses in preparation for liver surgery in patients with complicated liver anatomy and/or demanding surgical procedures including living-related liver transplantation [4].

**Figure 3:**
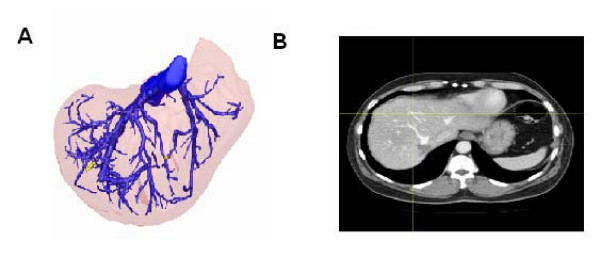
**A: 3D visualization of an intrahepatic shunt between the distal part of the middle hepatic vein and the right and left hepatic veins.** Since both CT images and 3D images are based on the same data set, every volume element (voxel) is uniquely defined in all three dimensions (x-, y- and z-axes). Our operations planning system allows us to identify the exact same voxel in both representations, i.e. in CT scans and 3D visualization. Here, the arrow directs to the yellow dot that is set as the original landmark in the 3D image. **B:** The crosshairs point to the exact same position as the yellow dot shown in figure 3A, indicating the position of the identical intrahepatic anastomosis in the CT image. One can speculate whether this vessel structure would have been correctly identified as an intravenous anastomosis if only the CT rather than the 3D images had been available.

The 3D images of the here presented data showed a missing middle hepatic vein (Figure 3A). Interestingly, the distal branches of the middle hepatic vein were still open and served as the origin of intervenous anastomoses to both the left and right hepatic veins (Figure 3B). Overall, six IVA were detectable, two to the left hepatic vein and four to the right hepatic vein. Because the proximal part of the middle hepatic vein was not detectable intraoperatively, one could speculate that the anatomical situation represents an anatomical variation rather than an after-effect of an old thrombosis.

Even though the CT images were sufficient to detect IVA, the 3D visualization improved the ability to detect IVA (Figures 3A and 3B), especially by using the features of the relocalization tool (description in Materials and Method section) which enables parallel searching for IVA in the CT scan and the 3D visualization. This parallel approach enabled us to detect intrahepatic anastomoses which could have been missed if only two-dimensional CT scans had been examined (figures 3A and 3B). Further, within the freely movable 3D images, the IVA could be detected very easily and fast. In contrast, the determination of IVA is time consuming with two-dimensional CT scans because one has to go back and forth within the image slices to follow the course of the IVA in order to find their drainage vessels.

The here presented data suggests that the information offered by 3D visualization is not redundant but rather serves as a true source of additional information especially in patients with complicated liver anatomy.

## Consent

Written informed consent was obtained from the patient for publication of this case report and any accompanying images. A copy of the written consent is available for review by the Editor-in-Chief of this journal.

## Competing interests

The authors hereby confirm that there exists no competing interest regarding their personal or financial relationship with other people or organizations. The authors disclose any financial competing interests but also any non-financial competing interests that may cause them embarrassment were they to become public after the publication of the manuscript.

## Authors' contributions

LF and BMS both conceived of the case report and drafted the manuscript. MS and H-PM post processed the image data to 3D images. SM and JON collected the patient's data and patients informed consent. MWB was involved in the planning of the study and the critical revision of the manuscript for important intellectual content. All authors read and approved the final manuscript.
